# Intentional Naproxen Overdose Causing Metabolic Acidosis: A Case Report

**DOI:** 10.7759/cureus.103545

**Published:** 2026-02-13

**Authors:** Brianne Falatovich, Blake Isom

**Affiliations:** 1 Emergency Medicine, Conemaugh Memorial Medical Center, Johnstown, USA; 2 Emergency Medicine, UPMC (University of Pittsburgh Medical Center) Harrisburg, Harrisburg, USA

**Keywords:** metabolic acidosis, multidrug, naproxen, nsaid, overdose

## Abstract

Naproxen is a widely available, over-the-counter medication often perceived as safe when used appropriately. However, in cases of overdose, especially when co-ingested with other substances, it may contribute to significant metabolic disturbances that are not commonly seen in nonsteroidal anti-inflammatory drug (NSAID) use.

We describe the case of a 24-year-old female who presented to the Emergency Department after an intentional multidrug overdose involving naproxen, hydroxyzine, and clonazepam. On arrival, she was found to have an elevated anion gap metabolic acidosis. Common causes of metabolic acidosis were ruled out, and her condition improved with supportive care, including intravenous fluids and a bicarbonate infusion. Clinical findings suggested that naproxen played a significant role in the development of the acid-base imbalance. This case report aims to illustrate that large doses of naproxen may contribute to metabolic acidosis in overdose situations. In cases of intentional ingestions involving multiple substances, clinicians should consider NSAIDs as potential contributors to acid-base abnormalities, particularly when more common causes are excluded.

## Introduction

Naproxen is a widely used nonsteroidal anti-inflammatory drug (NSAID), commonly indicated for the treatment of pain, inflammation, and fever. It acts via nonselective inhibition of cyclooxygenase (COX) enzymes--both COX‑1 and COX‑2--leading to reduced prostaglandin synthesis and inflammation suppression [1,2].

Though generally well-tolerated at therapeutic doses, naproxen can cause toxicity when consumed in large amounts. Adverse effects range from gastrointestinal irritation and bleeding to renal impairment, central nervous system depression, and, rarely, seizures or cardiovascular compromise [1-4]. Naproxen’s toxic potential is amplified by its pharmacokinetics, particularly its long half-life (12-17 hours) and high degree of protein binding (approximately 99%), which limit renal elimination and may prolong toxicity in overdose [1,2].

While NSAID toxicity is not classically associated with acid-base disturbances, elevated anion gap metabolic acidosis (AGMA) has been observed in rare cases of massive ingestion or when other toxic agents are co-ingested [4,5]. AGMA occurs when excess unmeasured acids accumulate in the bloodstream, leading to a decrease in serum bicarbonate and a widened anion gap, often reflecting an underlying pathology. In particular, patients with multidrug overdoses may present with a wide differential diagnosis for AGMA, including lactic acidosis, 5-oxoproline, or renal failure, all of which may be exacerbated by the presence of naproxen [3,5]. The accumulation of acidic metabolites in overdose is also a postulated mechanism for metabolic acidosis [6]. Previous literature linking naproxen overdose to AGMA is limited.

We present the case of a 24-year-old female who arrived at the emergency department following an intentional multidrug overdose and was found to have an elevated anion gap metabolic acidosis. This case report aims to illustrate that large doses of naproxen may contribute to metabolic acidosis in overdose situations. Although NSAIDs are not typically primary agents in acid-base disturbances, naproxen played a clinically relevant role in her presentation and biochemical derangement.

## Case presentation

A 24-year-old, 85.7 kg female presented to the Emergency Department (ED) following an intentional multi-drug overdose. She had a past medical history of depression, anxiety, a prior suicide attempt, ADHD, and borderline personality disorder. The patient reported that at approximately 0800 hours of the morning of her presentation, she ingested approximately 100 capsules of 220 mg naproxen (256.7 mg/kg), and an unknown amount of 10 mg hydroxyzine, 25 mg hydroxyzine, and 0.5 mg clonazepam. Other medications prescribed to her include lamotrigine and prazosin, though she had not ingested these. She presented to the ED at 0836. Initial vital signs in the ED were: temperature 98.8 °F, heart rate 127/min, respiratory rate 18/min, blood pressure 95/56 mmHg, and pulse oximetry saturation 97% on room air.

Her initial assessment was notable for nausea, abdominal pain, dizziness, headache, weakness, and blurred vision. She was not vomiting, agitated, or confused. On physical examination, she was awake and alert with a normal heart rate and rhythm. Pulmonary effort and breath sounds were normal. The abdomen was tender, but soft and flat. The skin was warm and dry. She had no focal neurological deficits. Her behavior was cooperative, and she had normal attention and speech. She exhibited a depressed mood and flat affect, with thought content including suicidal ideation and a plan.

At 0940 hours, a comprehensive ingestion workup was initiated, including a comprehensive metabolic panel, salicylate and acetaminophen levels, creatine kinase, magnesium, phosphorus, serum osmolality, complete blood count with differential, ethanol level, thyroid-stimulating hormone with reflex free T4, and a qualitative serum pregnancy test. This broad panel was obtained early to assess for metabolic derangements, occult co-ingestions, and end-organ injury.

Symptomatic management was initiated at 1019 hours, with the administration of sucralfate and ondansetron for abdominal discomfort and nausea. A 12-lead electrocardiogram was obtained at 1032 hours for medical clearance and evaluation of potential cardiotoxic effects of co-ingested agents, which did not reveal acute abnormalities.

At 1048 hours, the patient received a 1-liter bolus of 0.9% sodium chloride for initial hypotension and tachycardia. A point-of-care urine pregnancy test was completed at 1049 hours, followed by a urine drug screen and urinalysis at 1057 hours to assess for substances not captured on serum testing.

At 1106 hours, a venous blood gas was obtained, revealing a primary metabolic acidosis with an elevated anion gap, prompting further evaluation for causes of anion gap metabolic acidosis in the setting of overdose. A serum lactic acid level was drawn at 1204 hours and was within normal limits, effectively excluding lactic acidosis as the primary etiology. These pertinent laboratory values can be seen in Table 1.

**Table 1:** Pertinent emergency department laboratory studies, demonstrating anion gap metabolic acidosis pCO2: partial pressure of carbon dioxide; pO2: partial pressure of oxygen; HCO3: bicarbonate; eGFR: estimated glomerular filtration rate

Parameter	Reference Level	Value
Urine Drug Screen (UDS)		
Amphetamines	None Detected	None Detected
Barbiturates	None Detected	None Detected
Benzodiazepines	None Detected	None Detected
Cannabinoids	None Detected	Detected
Cocaine Screen	None Detected	None Detected
Methadone	None Detected	None Detected
Opiate Screen	None Detected	None Detected
Fentanyl Screen	None Detected	None Detected
Oxycodone	None Detected	None Detected
Buprenorphine	None Detected	None Detected
Other Drug Levels		
Ethanol Level	<10	<10
Acetaminophen	<10	<10
Salicylate Level	<3	<3
Venous Blood Gas		
Venous pH	7.32-7.42	7.22
Venous pCO2	37-47	37
pO2 Venous	30-40	57
HCO3 Venous	21-28	15
Venous O2	94-100	84
Anion Gap	2-12	18
Renal Function		
Blood Urea Nitrogen	5-20	5
Creatinine	0.5-1.1	0.63
eGFR	>90	127

Given the presence of unexplained AGMA and the ongoing need for monitoring, the patient was placed in observation status at 1209 hours. Toxicology was consulted shortly thereafter to assist with the interpretation of the acid-base disturbance and management of reported co-ingestions.

At 1304 hours, additional laboratory testing was obtained, including prothrombin time-international normalized ratio (INR) and a lamotrigine level, given the patient’s prescription history. The lamotrigine level was subtherapeutic, supporting the patient’s report that it was not ingested in a significant quantity. Due to metabolic acidosis, a sodium bicarbonate infusion (150 mEq in D5W at 125 mL/hr) was initiated at 1304 hours. Scheduled home lamotrigine was administered concurrently to avoid withdrawal or mood destabilization.

Given the reported co-ingestion of hydroxyzine and clonazepam, additional toxicologic considerations were addressed. Hydroxyzine toxicity can manifest as an antimuscarinic toxidrome, including tachycardia, agitation, urinary retention, and encephalopathy. The patient did not exhibit these findings on serial examinations. Toxicology recommended supportive care and benzodiazepines for agitation if needed, with bladder scanning if agitation were refractory, to assess for urinary retention as a precipitating factor. As these features did not develop, clinically significant hydroxyzine toxicity was considered unlikely.

Clonazepam ingestion was also considered as a contributor to altered mental status. Routine urine drug screens may not detect clonazepam due to assay limitations, and, therefore, a negative benzodiazepine screen did not exclude exposure. However, isolated clonazepam overdose is typically benign, with sedation being the predominant clinical effect. The patient did not demonstrate significant respiratory depression or progressive encephalopathy.

A repeat salicylate level was obtained at 1646, per toxicology recommendations, to account for possible delayed absorption or unreliable ingestion history. This level remained undetectable, definitively excluding salicylate toxicity as a cause of the metabolic acidosis.

With common causes of AGMA excluded, attention turned to the patient’s reported naproxen ingestion. While NSAIDs are not classically associated with significant acid-base disturbances, rare cases of AGMA have been described following massive ingestion ranging between 20g and 70g [4,6]. Proposed mechanisms include accumulation of organic acids, mitochondrial dysfunction, and impaired renal acid excretion, particularly in the setting of large doses [6].

In this patient, the absence of alternative explanations, normal lactate and osmolar gap, preserved renal function, and temporal association with a massive naproxen ingestion supported naproxen toxicity as the most likely etiology of her AGMA. Toxicology consultation concurred with this assessment and recommended close monitoring for progression to lactic acidosis, acute kidney injury, seizures, and worsening encephalopathy.

The patient remained hemodynamically stable throughout the afternoon and evening. At 1842 hours, she received lorazepam for anxiety without evidence of worsening encephalopathy or respiratory depression. A second sodium bicarbonate infusion was initiated at 2125 hours due to ongoing but improving metabolic acidosis.

The following morning, repeat laboratory testing at 0517 hours demonstrated resolution of the anion gap metabolic acidosis, with normalization of bicarbonate levels and continued stable renal function. A final sodium bicarbonate infusion was administered at 0635 hours, and supportive inpatient medications, including enoxaparin for venous thromboembolism prophylaxis and potassium repletion, were given later that morning. With sustained clinical stability, resolution of metabolic abnormalities, and no evidence of delayed toxicity or organ dysfunction, the patient was medically cleared and discharged in 2005 for transfer to a psychiatric facility under voluntary commitment.

This timeline of the case highlights the prompt identification of metabolic acidosis, early exclusion of common toxicologic causes of AGMA through targeted laboratory evaluation, and appropriate escalation to toxicology consultation. Serial testing, particularly repeat salicylate levels and metabolic panels, was critical in excluding delayed co-ingestions and confirming naproxen-induced metabolic acidosis as the most likely etiology. Early supportive care and bicarbonate therapy resulted in full metabolic resolution within 24 hours.

## Discussion

This case highlights the diagnostic and therapeutic challenges associated with multidrug overdose, particularly when complicated by AGMA. While AGMA is a common finding in toxicologic presentations, NSAIDs such as naproxen are not frequently recognized as contributors. However, in the context of massive ingestion or co-ingestion with other agents, naproxen may exacerbate or indirectly cause metabolic derangements.

The differential diagnosis for AGMA in overdose scenarios includes various well-known etiologies such as lactic acidosis, ketoacidosis, renal failure, and ingestion of toxic alcohols or salicylates. Additionally, less common causes like 5-oxoproline (pyroglutamic acid) accumulation, typically associated with acetaminophen toxicity, must also be considered in polypharmacy cases [5]. In this patient, although multiple substances were reportedly ingested, naproxen emerged as a clinically relevant agent due to the timing of the acidosis, its pharmacologic profile, and its potential to impair renal function.

Naproxen is known for its high protein-binding capacity (approximately 99%) and relatively long half-life, ranging from 12 to 17 hours [1]. In overdose, the saturation of protein-binding sites results in increased levels of free drug, which can enhance toxicity [1]. Furthermore, the drug’s extended half-life and potential for enterohepatic recirculation contribute to prolonged systemic exposure and may delay clinical recovery [1]. While AGMA is not a classical feature of NSAID toxicity, case reports have documented its occurrence, particularly when large doses are ingested or renal function becomes impaired [4].

Renal injury is a key mechanism by which naproxen can contribute to acid-base abnormalities [2]. NSAIDs inhibit prostaglandin synthesis, which reduces renal blood flow and can precipitate acute kidney injury (AKI), particularly in volume-depleted or predisposed individuals [2-4]. AKI, in turn, limits the kidneys' ability to excrete hydrogen ions and organic acids, potentially leading to or worsening AGMA [3]. Although in this case the complete panel of co-ingestants and toxin levels was not available, naproxen likely played a significant role in the development or persistence of metabolic acidosis, as common causes were appropriately ruled out.

Moreover, the presence of co-ingestants can interact with naproxen’s effects and complicate the clinical picture. For example, acetaminophen, when taken in overdose, may lead to pyroglutamic acidosis through glutathione depletion and disruption of the gamma-glutamyl cycle [5]. If sedatives, opioids, or antidepressants are co-ingested, as in this case, they may contribute to respiratory depression, lactic acidosis, or even seizures, further altering acid-base balance. This overlap makes it difficult to attribute AGMA to a single agent, underscoring the importance of comprehensive diagnostic workup and serial laboratory monitoring [7].

Management of naproxen overdose is largely supportive. Activated charcoal may be administered within a few hours of ingestion, particularly given naproxen’s delayed absorption and enterohepatic cycling [1]. However, the role of activated charcoal is uncertain, given time constraints and unclear benefits [2]. While hemodialysis is generally not effective due to the drug’s high protein binding, supportive measures, such as intravenous fluids, electrolyte correction, and close renal monitoring, are essential. In rare cases where renal failure or severe acidosis develops, consultation with nephrology or toxicology may be warranted to consider extracorporeal treatments, although evidence for their efficacy in NSAID toxicity remains limited [4].

This case also illustrates the potential utility of measuring serum naproxen concentrations when available. While not measured in this case, serial drug levels can offer insight into pharmacokinetics, absorption patterns, and correlate with clinical findings, as demonstrated in previous reports of massive naproxen overdose [4,6]. While such testing is not routine, it may be helpful in severe cases, especially when acid-base disturbances or prolonged symptoms occur. It should be noted that measuring serum naproxen levels may provide limited benefit to patient care, as results may not be available on an expedited basis, which limits its utility in the acute setting.

In summary, although naproxen is not typically associated with AGMA, this case emphasizes its potential to contribute to metabolic acidosis in the setting of intentional multidrug ingestion. Clinicians should maintain a broad differential diagnosis for AGMA in overdose patients and recognize that NSAIDs, particularly in large doses or with renal involvement, may be significant contributors. Serial lab evaluation, supportive management, and awareness of less common toxicity mechanisms are essential to optimizing patient outcomes. 

## Conclusions

Although naproxen is not classically associated with elevated anion gap metabolic acidosis, this case demonstrates that it may contribute to such disturbances when taken in large quantities. Its pharmacologic properties and potential effects on renal function can compound the clinical picture in multidrug overdose scenarios. In patients with unexplained acidosis following overdose, NSAID toxicity should be considered in the differential, even when not initially suspected. Prompt supportive management and monitoring are essential to guide recovery.
